# 4-Fluoro-*N*-[3-(2-fluoro­phen­yl)-4-methyl-2,3-dihydro-2-thienyl­idene]benzamide

**DOI:** 10.1107/S1600536809022314

**Published:** 2009-06-20

**Authors:** Aamer Saeed, Uzma Shaheen, Muhammad Latif, Michael Bolte

**Affiliations:** a Department of Chemistry, Quaid-i-Azam University, Islamabad 45320, Pakistan; b Hamdard Institute of Pharmaceutical Sciences, Hamdard University, Islamabad Campus, Pakistan; c Institut für Anorganische Chemie, J. W. Goethe-Universität Frankfurt, Max-von-Laue-Strasse 7, 60438 Frankfurt/Main, Germany

## Abstract

In the title compound, C_17_H_12_F_2_N_2_OS, the planar thia­zole ring (r.m.s. deviation = 0.012 Å) makes dihedral angles of 15.08 (9) and 81.81 (6)° with the 4-fluoro­phenyl and 2-fluoro­phenyl rings, respectively. The 2-fluoro­phenyl ring is disordered over two orientations with site-occupancy factors of 0.810 (3) and 0.190 (3). The structure contains inter­molecular C—H⋯O hydrogen bonds.

## Related literature

For the biological activity of imino-1,3-thia­zoline derivatives, see: Kim *et al.* (2007 ▶); Vicini *et al.* (2006 ▶); Hosseinimehr *et al.* (2001 ▶); Zhang *et al.* (2000 ▶); Pietrancosta *et al.* (2006 ▶). For details of the synthesis, see: Saeed *et al.* (2008*a* ▶). For a related structure, see: Saeed *et al.* (2008*b* ▶).
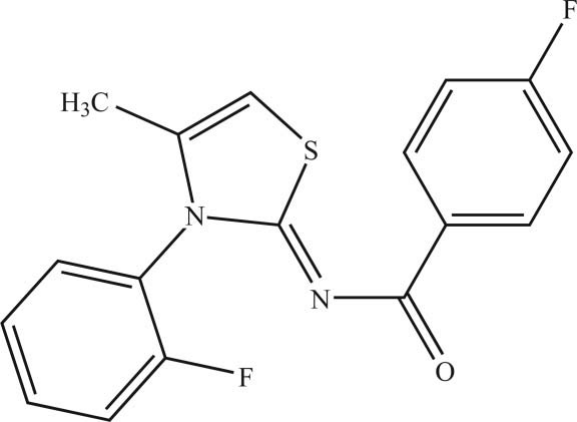


## Experimental

### 

#### Crystal data

C_17_H_12_F_2_N_2_OS
*M* *_r_* = 330.35
Orthorhombic, 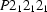

*a* = 7.0982 (14) Å
*b* = 11.423 (2) Å
*c* = 18.949 (4) Å
*V* = 1536.5 (5) Å^3^
*Z* = 4
Mo *K*α radiation
μ = 0.24 mm^−1^
*T* = 173 K
0.36 × 0.34 × 0.28 mm

#### Data collection

Stoe IPDS-II two-circle diffractometer
Absorption correction: multi-scan (*MULABS*; Spek, 2009 ▶; Blessing, 1995 ▶) *T*_min_ = 0.920, *T*_max_ = 0.937
10484 measured reflections
3531 independent reflections
3213 reflections with *I* > 2σ(*I*)
*R*_int_ = 0.046

#### Refinement

*R*[*F* ^2^ > 2σ(*F* ^2^)] = 0.034
*wR*(*F* ^2^) = 0.082
*S* = 0.99
3531 reflections
219 parameters
H-atom parameters constrained
Δρ_max_ = 0.15 e Å^−3^
Δρ_min_ = −0.26 e Å^−3^
Absolute structure: Flack (1983 ▶), 1491 Friedel pairs
Flack parameter: −0.15 (6)

### 

Data collection: *X-AREA* (Stoe & Cie, 2001 ▶); cell refinement: *X-AREA*; data reduction: *X-AREA*; program(s) used to solve structure: *SHELXS97* (Sheldrick, 2008 ▶); program(s) used to refine structure: *SHELXL97* (Sheldrick, 2008 ▶); molecular graphics: *SHELXTL* (Sheldrick, 2008 ▶); software used to prepare material for publication: *SHELXTL*.

## Supplementary Material

Crystal structure: contains datablocks global, I. DOI: 10.1107/S1600536809022314/bi2375sup1.cif

Structure factors: contains datablocks I. DOI: 10.1107/S1600536809022314/bi2375Isup2.hkl

Additional supplementary materials:  crystallographic information; 3D view; checkCIF report

## Figures and Tables

**Table 1:** Hydrogen-bond geometry (Å, °)

*D*—H⋯*A*	*D*—H	H⋯*A*	*D*⋯*A*	*D*—H⋯*A*
C5—H5⋯O1^i^	0.95	2.41	3.322 (2)	160

Symmetry code: (i) 
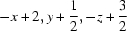
.

**Fig. 1.**
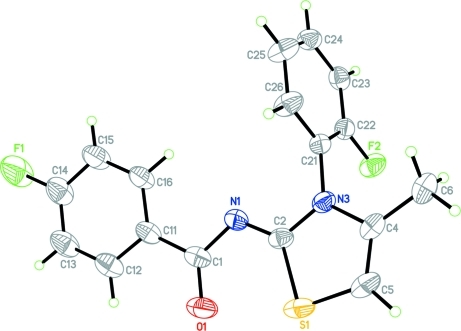
Perspective view of the title compound with the atom numbering scheme; displacement ellipsoids are at the 50% probability level; H atoms are drawn as small spheres of arbitrary radii. The atoms of the minor occupied sites have been omitted for clarity.

## supplementary crystallographic information

### Comment

The imino-1,3-thiazoline group is found in a variety of biologically active natural products and finds extensive applications in medicinal chemistry. 2-Thiazolylimino-5-arylidene-4-thiazolidinones show noticeable antimicrobial activity against bacteria, yeasts and mould (Kim *et al.*, 2007). 3-Substituted 2-(cyanoimino)thiazolidines can be used in agriculture due to their neonicotinoid insecticidal activity (Vicini *et al.*, 2006). 3-Substituted thiazolidines show radioprotective properties against γ-radiation (Hosseinimehr *et al.*, 2001). KHG22394, a 2-imino-1,3-thiazoline derivative, significantly inhibits melanin production in a dose-dependent manner, thus acting as a skin whitening agent (Zhang *et al.*, 2000) and pifithrin-alpha, another iminothiazoline, is a reversible inhibitor of p53-mediated apoptosis and p53-dependent gene transcription (Pietrancosta *et al.*, 2006).

### Experimental

The title compound was prepared according to the procedure reported earlier (Saeed *et al.* (2008*a*). Crystallization of the residue in CHCl_3_ afforded the title compound (81%) as white needles: Anal. calcd. for C_14_H_12_Cl_NO_1: C 68.44, H 4.92, N 5.70%; found: C 68.39, H 4.90, N 5.67%

### Refinement

H atoms were geometrically positioned and refined using a riding model with fixed individual displacement parameters [U_iso_(H) = 1.2 *U*_eq_(C) or 1.5 *U*_eq_(C_methyl_)] using a riding model with C—H(aromatic) = 0.95 Å or C—H(methyl) = 0.98 Å. The *ortho*-fluoro-phenyl ring is disordered over two positions with site occupation factors of 0.810 (3) and 0.190 (3).

### Crystal data

**Table:**

C_17_H_12_F_2_N_2_OS	*F*(000) = 680
*M**_r_* = 330.35	*D*_x_ = 1.428 Mg m^−^^3^
Orthorhombic, *P*2_1_2_1_2_1_	Mo *K*α radiation, λ = 0.71073 Å
Hall symbol: P 2ac 2ab	Cell parameters from 9675 reflections
*a* = 7.0982 (14) Å	θ = 3.4–27.8°
*b* = 11.423 (2) Å	µ = 0.24 mm^−^^1^
*c* = 18.949 (4) Å	*T* = 173 K
*V* = 1536.5 (5) Å^3^	Block, colourless
*Z* = 4	0.36 × 0.34 × 0.28 mm

### Data collection

**Table:**

Stoe IPDS-II two-circle diffractometer	3531 independent reflections
Radiation source: fine-focus sealed tube	3213 reflections with *I* > 2σ(*I*)
graphite	*R*_int_ = 0.046
ω scans	θ_max_ = 27.6°, θ_min_ = 3.4°
Absorption correction: multi-scan (*MULABS*; Spek, 2009; Blessing, 1995)	*h* = −8→9
*T*_min_ = 0.920, *T*_max_ = 0.937	*k* = −12→14
10484 measured reflections	*l* = −24→23

### Refinement

**Table:**

Refinement on *F*^2^	Secondary atom site location: difference Fourier map
Least-squares matrix: full	Hydrogen site location: inferred from neighbouring sites
*R*[*F*^2^ > 2σ(*F*^2^)] = 0.034	H-atom parameters constrained
*wR*(*F*^2^) = 0.082	*w* = 1/[σ^2^(*F*_o_^2^) + (0.052*P*)^2^] where *P* = (*F*_o_^2^ + 2*F*_c_^2^)/3
*S* = 0.99	(Δ/σ)_max_ = 0.001
3531 reflections	Δρ_max_ = 0.15 e Å^−^^3^
219 parameters	Δρ_min_ = −0.26 e Å^−^^3^
0 restraints	Absolute structure: Flack (1983), 1491 Friedel pairs
Primary atom site location: structure-invariant direct methods	Flack parameter: −0.15 (6)

### Special details

**Table:**

Geometry. All e.s.d.'s (except the e.s.d. in the dihedral angle between two l.s. planes) are estimated using the full covariance matrix. The cell e.s.d.'s are taken into account individually in the estimation of e.s.d.'s in distances, angles and torsion angles; correlations between e.s.d.'s in cell parameters are only used when they are defined by crystal symmetry. An approximate (isotropic) treatment of cell e.s.d.'s is used for estimating e.s.d.'s involving l.s. planes.
Refinement. Refinement of *F*^2^ against ALL reflections. The weighted *R*-factor *wR* and goodness of fit *S* are based on *F*^2^, conventional *R*-factors *R* are based on *F*, with *F* set to zero for negative *F*^2^. The threshold expression of *F*^2^ > σ(*F*^2^) is used only for calculating *R*-factors(gt) *etc*. and is not relevant to the choice of reflections for refinement. *R*-factors based on *F*^2^ are statistically about twice as large as those based on *F*, and *R*- factors based on ALL data will be even larger.

### Fractional atomic coordinates and isotropic or equivalent isotropic displacement parameters (Å^2^)

**Table:**

	*x*	*y*	*z*	*U*_iso_*/*U*_eq_	Occ. (<1)
S1	0.86255 (5)	0.65177 (5)	0.73205 (2)	0.04103 (12)	
N1	0.56925 (19)	0.59033 (13)	0.64274 (7)	0.0316 (3)	
O1	0.7594 (2)	0.44060 (14)	0.68401 (8)	0.0520 (4)	
F1	0.0840 (3)	0.15356 (14)	0.55262 (9)	0.0825 (5)	
F2	0.71228 (15)	0.86728 (14)	0.53579 (6)	0.0406 (4)	0.810 (3)
F2'	0.2818 (7)	0.7551 (6)	0.7021 (3)	0.048 (2)	0.190 (3)
C1	0.6168 (2)	0.47540 (16)	0.65201 (8)	0.0356 (4)	
C2	0.67222 (19)	0.66920 (16)	0.67514 (8)	0.0303 (3)	
N3	0.63342 (18)	0.78545 (13)	0.66789 (7)	0.0304 (3)	
C4	0.7456 (2)	0.86166 (18)	0.70871 (8)	0.0340 (4)	
C5	0.8742 (2)	0.80204 (19)	0.74587 (9)	0.0414 (4)	
H5	0.9626	0.8381	0.7766	0.050*	
C6	0.7117 (3)	0.98996 (19)	0.70565 (10)	0.0436 (4)	
H6A	0.7834	1.0287	0.7432	0.065*	
H6B	0.5771	1.0056	0.7121	0.065*	
H6C	0.7523	1.0201	0.6597	0.065*	
C11	0.4788 (3)	0.39042 (17)	0.62212 (8)	0.0353 (4)	
C12	0.5145 (3)	0.27036 (19)	0.62624 (10)	0.0451 (4)	
H12	0.6301	0.2435	0.6457	0.054*	
C13	0.3827 (3)	0.19005 (18)	0.60216 (10)	0.0513 (5)	
H13	0.4062	0.1083	0.6052	0.062*	
C14	0.2173 (3)	0.2315 (2)	0.57387 (11)	0.0529 (5)	
C15	0.1774 (3)	0.3489 (2)	0.56784 (11)	0.0519 (5)	
H15	0.0622	0.3747	0.5476	0.062*	
C16	0.3098 (3)	0.42878 (17)	0.59203 (10)	0.0405 (4)	
H16	0.2854	0.5103	0.5881	0.049*	
C21	0.48714 (19)	0.82453 (15)	0.62105 (8)	0.0278 (3)	
C22	0.5303 (2)	0.86227 (15)	0.55392 (8)	0.0290 (3)	
H22	0.6587	0.8661	0.5400	0.035*	0.190 (3)
C23	0.3925 (2)	0.89464 (15)	0.50637 (8)	0.0325 (3)	
H23	0.4250	0.9201	0.4602	0.039*	
C24	0.2054 (2)	0.88918 (16)	0.52746 (9)	0.0348 (4)	
H24	0.1085	0.9107	0.4954	0.042*	
C25	0.1592 (2)	0.8527 (2)	0.59472 (9)	0.0439 (4)	
H25	0.0309	0.8499	0.6089	0.053*	
C26	0.2997 (2)	0.8203 (2)	0.64164 (9)	0.0403 (4)	
H26	0.2675	0.7945	0.6877	0.048*	0.810 (3)

### Atomic displacement parameters (Å^2^)

**Table:**

	*U*^11^	*U*^22^	*U*^33^	*U*^12^	*U*^13^	*U*^23^
S1	0.02961 (17)	0.0565 (3)	0.0369 (2)	0.00724 (19)	−0.00870 (15)	0.0061 (2)
N1	0.0334 (6)	0.0324 (8)	0.0290 (6)	0.0029 (5)	−0.0029 (5)	0.0041 (6)
O1	0.0474 (7)	0.0475 (9)	0.0610 (8)	0.0136 (6)	−0.0158 (6)	0.0073 (7)
F1	0.1076 (11)	0.0365 (7)	0.1034 (11)	−0.0068 (8)	−0.0504 (9)	−0.0073 (8)
F2	0.0270 (6)	0.0573 (10)	0.0374 (6)	−0.0039 (5)	0.0046 (4)	0.0079 (6)
F2'	0.032 (3)	0.074 (5)	0.038 (3)	0.005 (3)	0.006 (2)	0.014 (3)
C1	0.0391 (8)	0.0375 (9)	0.0302 (7)	0.0106 (8)	−0.0003 (6)	0.0046 (7)
C2	0.0263 (6)	0.0403 (10)	0.0242 (6)	0.0023 (6)	−0.0005 (5)	0.0036 (6)
N3	0.0260 (5)	0.0362 (7)	0.0290 (6)	−0.0027 (6)	−0.0054 (5)	0.0023 (5)
C4	0.0269 (6)	0.0454 (11)	0.0299 (7)	−0.0076 (7)	−0.0004 (5)	−0.0033 (7)
C5	0.0288 (7)	0.0603 (12)	0.0351 (8)	−0.0059 (7)	−0.0074 (6)	−0.0013 (7)
C6	0.0402 (8)	0.0462 (12)	0.0443 (9)	−0.0112 (8)	−0.0037 (7)	−0.0079 (8)
C11	0.0460 (9)	0.0325 (9)	0.0275 (7)	0.0087 (7)	−0.0001 (6)	0.0021 (6)
C12	0.0590 (11)	0.0372 (10)	0.0391 (9)	0.0129 (9)	−0.0036 (8)	−0.0004 (8)
C13	0.0751 (13)	0.0302 (10)	0.0485 (10)	0.0111 (9)	−0.0098 (10)	−0.0064 (8)
C14	0.0751 (13)	0.0332 (11)	0.0504 (11)	−0.0010 (10)	−0.0168 (10)	−0.0057 (9)
C15	0.0656 (11)	0.0348 (10)	0.0553 (11)	0.0055 (9)	−0.0237 (9)	−0.0007 (10)
C16	0.0525 (10)	0.0295 (9)	0.0395 (8)	0.0064 (7)	−0.0111 (7)	0.0015 (8)
C21	0.0262 (6)	0.0285 (8)	0.0288 (7)	−0.0004 (6)	−0.0037 (5)	0.0016 (6)
C22	0.0280 (6)	0.0278 (8)	0.0314 (7)	−0.0019 (6)	0.0010 (5)	0.0002 (6)
C23	0.0409 (8)	0.0299 (8)	0.0267 (7)	−0.0024 (7)	−0.0022 (6)	0.0021 (6)
C24	0.0335 (7)	0.0363 (9)	0.0346 (8)	0.0029 (7)	−0.0097 (6)	−0.0014 (7)
C25	0.0258 (6)	0.0679 (13)	0.0379 (8)	0.0020 (8)	−0.0020 (6)	0.0010 (9)
C26	0.0290 (7)	0.0615 (13)	0.0305 (7)	−0.0027 (8)	−0.0009 (6)	0.0070 (8)

### Geometric parameters (Å, °)

**Table:**

S1—C5	1.738 (2)	C12—C13	1.388 (3)
S1—C2	1.7400 (15)	C12—H12	0.950
N1—C2	1.313 (2)	C13—C14	1.375 (3)
N1—C1	1.367 (2)	C13—H13	0.950
O1—C1	1.245 (2)	C14—C15	1.376 (3)
F1—C14	1.360 (3)	C15—C16	1.387 (3)
F2—C22	1.3379 (18)	C15—H15	0.950
F2'—C26	1.373 (6)	C16—H16	0.950
C1—C11	1.491 (3)	C21—C22	1.378 (2)
C2—N3	1.363 (2)	C21—C26	1.387 (2)
N3—C4	1.411 (2)	C22—C23	1.380 (2)
N3—C21	1.4370 (18)	C22—H22	0.950
C4—C5	1.339 (2)	C23—C24	1.388 (2)
C4—C6	1.486 (3)	C23—H23	0.950
C5—H5	0.950	C24—C25	1.380 (2)
C6—H6A	0.980	C24—H24	0.950
C6—H6B	0.980	C25—C26	1.386 (2)
C6—H6C	0.980	C25—H25	0.950
C11—C12	1.397 (3)	C26—H26	0.950
C11—C16	1.399 (2)		
			
C5—S1—C2	90.99 (8)	F1—C14—C13	119.0 (2)
C2—N1—C1	117.49 (14)	F1—C14—C15	118.1 (2)
O1—C1—N1	124.75 (18)	C13—C14—C15	122.9 (2)
O1—C1—C11	120.75 (17)	C14—C15—C16	118.31 (19)
N1—C1—C11	114.48 (14)	C14—C15—H15	120.8
N1—C2—N3	120.60 (13)	C16—C15—H15	120.8
N1—C2—S1	130.07 (14)	C15—C16—C11	120.64 (18)
N3—C2—S1	109.32 (12)	C15—C16—H16	119.7
C2—N3—C4	115.58 (14)	C11—C16—H16	119.7
C2—N3—C21	120.72 (13)	C22—C21—C26	118.93 (14)
C4—N3—C21	123.70 (15)	C22—C21—N3	120.45 (13)
C5—C4—N3	111.06 (17)	C26—C21—N3	120.56 (14)
C5—C4—C6	129.23 (16)	F2—C22—C21	117.72 (14)
N3—C4—C6	119.71 (15)	F2—C22—C23	120.34 (14)
C4—C5—S1	113.00 (13)	C21—C22—C23	121.94 (14)
C4—C5—H5	123.5	C21—C22—H22	118.9
S1—C5—H5	123.5	C23—C22—H22	119.1
C4—C6—H6A	109.5	C22—C23—C24	118.55 (15)
C4—C6—H6B	109.5	C22—C23—H23	120.7
H6A—C6—H6B	109.5	C24—C23—H23	120.7
C4—C6—H6C	109.5	C25—C24—C23	120.43 (14)
H6A—C6—H6C	109.5	C25—C24—H24	119.8
H6B—C6—H6C	109.5	C23—C24—H24	119.8
C12—C11—C16	119.08 (18)	C24—C25—C26	120.12 (14)
C12—C11—C1	119.91 (16)	C24—C25—H25	119.9
C16—C11—C1	120.96 (16)	C26—C25—H25	119.9
C13—C12—C11	120.54 (18)	F2'—C26—C25	127.8 (3)
C13—C12—H12	119.7	F2'—C26—C21	110.0 (3)
C11—C12—H12	119.7	C25—C26—C21	120.02 (15)
C14—C13—C12	118.5 (2)	C25—C26—H26	119.9
C14—C13—H13	120.8	C21—C26—H26	120.0
C12—C13—H13	120.8		
			
C2—N1—C1—O1	−6.3 (3)	C12—C13—C14—C15	0.6 (3)
C2—N1—C1—C11	171.87 (14)	F1—C14—C15—C16	177.5 (2)
C1—N1—C2—N3	179.61 (14)	C13—C14—C15—C16	−0.6 (4)
C1—N1—C2—S1	−2.0 (2)	C14—C15—C16—C11	−0.4 (3)
C5—S1—C2—N1	−176.30 (15)	C12—C11—C16—C15	1.3 (3)
C5—S1—C2—N3	2.19 (11)	C1—C11—C16—C15	−176.43 (18)
N1—C2—N3—C4	176.05 (13)	C2—N3—C21—C22	−97.29 (18)
S1—C2—N3—C4	−2.61 (15)	C4—N3—C21—C22	82.51 (19)
N1—C2—N3—C21	−4.1 (2)	C2—N3—C21—C26	80.0 (2)
S1—C2—N3—C21	177.21 (10)	C4—N3—C21—C26	−100.2 (2)
C2—N3—C4—C5	1.65 (19)	C26—C21—C22—F2	178.95 (18)
C21—N3—C4—C5	−178.16 (13)	N3—C21—C22—F2	−3.7 (2)
C2—N3—C4—C6	−178.75 (14)	C26—C21—C22—C23	−0.8 (3)
C21—N3—C4—C6	1.4 (2)	N3—C21—C22—C23	176.59 (16)
N3—C4—C5—S1	0.14 (17)	F2—C22—C23—C24	−179.43 (18)
C6—C4—C5—S1	−179.40 (14)	C21—C22—C23—C24	0.3 (3)
C2—S1—C5—C4	−1.36 (13)	C22—C23—C24—C25	0.4 (3)
O1—C1—C11—C12	−3.8 (3)	C23—C24—C25—C26	−0.5 (3)
N1—C1—C11—C12	177.96 (16)	C24—C25—C26—F2'	−161.8 (4)
O1—C1—C11—C16	173.91 (17)	C24—C25—C26—C21	0.0 (3)
N1—C1—C11—C16	−4.3 (2)	C22—C21—C26—F2'	165.4 (3)
C16—C11—C12—C13	−1.3 (3)	N3—C21—C26—F2'	−12.0 (4)
C1—C11—C12—C13	176.43 (16)	C22—C21—C26—C25	0.6 (3)
C11—C12—C13—C14	0.4 (3)	N3—C21—C26—C25	−176.76 (18)
C12—C13—C14—F1	−177.5 (2)		

### Hydrogen-bond geometry (Å, °)

**Table:**

*D*—H···*A*	*D*—H	H···*A*	*D*···*A*	*D*—H···*A*
C5—H5···O1^i^	0.95	2.41	3.322 (2)	160

Symmetry codes: (i) −*x*+2, *y*+1/2, −*z*+3/2.
